# Respiratory Interventions for Preterm Infants in LMICs: A Prospective Study From Cape Town, South Africa

**DOI:** 10.3389/fgwh.2022.817817

**Published:** 2022-04-06

**Authors:** Ilse Lategan, Caris Price, Natasha Raygaan Rhoda, Heather J. Zar, Lloyd Tooke

**Affiliations:** ^1^Department of Paediatrics and Child Health, University of Cape Town, Cape Town, South Africa; ^2^South African Medical Research Council (SA-MRC) Unit on Child and Adolescent Health, University of Cape Town, Cape Town, South Africa

**Keywords:** respiratory, interventions, preterm (birth), low-and-middle income countries (LMIC), continuous positive airway pressure (CPAP), surfactant, invasive mechanical ventilation, respiratory distress syndrome (RDS)

## Abstract

**Background:**

Preterm birth is a global public health issue and complications of preterm birth result in the death of approximately 1 million infants each year, 99% of which are in low-and-middle income countries (LMIC). Although respiratory interventions such as continuous positive airway pressure (CPAP) and surfactant have been shown to improve the outcomes of preterm infants with respiratory distress, they are not readily available in low-resourced areas. The aim of this study was to report the respiratory support needs and outcomes of preterm infants in a low-resourced setting, and to estimate the impact of a lack of access to these interventions on neonatal mortality.

**Methods:**

We conducted a six-month prospective observational study on preterm infants <1,801 g admitted at Groote Schuur Hospital and Mowbray Maternity Hospital neonatal units in Cape Town, South Africa. We extrapolated results from the study to model the potential outcomes of these infants in the absence of these interventions.

**Results:**

Five hundred and fifty-two infants (552) <1,801 g were admitted. Three hundred (54.3%) infants received CPAP, and this was the initial respiratory intervention for most cases of respiratory distress syndrome. Surfactant was given to 100 (18.1%) infants and a less invasive method was the most common method of administration. Invasive mechanical ventilation was offered to 105 (19%) infants, of which only 57 (54.2%) survived until discharge from hospital. The overall mortality of the cohort was 14.1% and the hypothetical removal of invasive mechanical ventilation, surfactant and CPAP would result in an additional 157 deaths and increase the overall mortality to 42.5%. A lack of CPAP availability would have the largest impact on mortality and result in the largest number of additional deaths (109).

**Conclusion:**

This study highlights the effect that access to key respiratory interventions has on preterm outcomes in LMICs. CPAP has the largest impact on neonatal mortality and improving its coverage should be the primary goal for low-resourced areas to save newborn lives.

## Introduction

Preterm birth is a global public health issue that complicates an estimated 14.8 million deliveries annually. Although there is significant variation in the rate of preterm births globally, low-and-middle income countries (LMICs) account for the majority of preterm births with over 81% of preterm births occurring in sub-Saharan Africa and Asia. Preterm birth accounts for 35% of all neonatal deaths and is the single largest direct cause of neonatal deaths resulting in the death of approximately one million infants each year of which over 99% are in LMICs (1–4).

In the absence of appropriate treatment, respiratory failure is a common mode of death from surfactant deficiency, apnoea, and infection. In well-resourced countries, neonatal respiratory care includes non-invasive ventilatory support methods such as nasal cannula oxygen, continuous positive airway pressure (CPAP) and heated humidified high flow oxygen (HHHF). Should escalation be required, intubation and conventional mechanical ventilation (CMV) or high frequency oscillatory ventilation (HFOV) can be delivered. Infants with respiratory distress syndrome (RDS) may benefit from early administration of exogenous surfactant. In spontaneously breathing infants intubation, surfactant administration and extubation (INSURE) and thin catheter administration/less invasive surfactant administration (TCA/LISA) may be performed in an effort to prevent MV. TCA/LISA, as compared to INSURE, does not require positive pressure breaths and has been shown to decrease bronchopulmonary dysplasia (BPD) (5).

Many LMICs, especially on the African continent, have inadequate access to respiratory care interventions for preterm infants. In 2020, only 63% of African countries had any access to CPAP and only 33% had access to surfactant replacement therapy (SRT). Further inequality is evident when comparing public to private hospitals and urban to rural areas; for example, only 28% of countries had access to CPAP in more than 10% of their cities, leaving most preterm infants without this effective intervention (6).

South Africa is an upper-middle income country, one of only eight such countries on the continent. Within South Africa, there is inequality in access to resources both geographically and between public and private hospital sectors. Whilst total healthcare expenditure is similar between the two sectors, only 20% of the population utilize the more well-resourced private healthcare system (7). These inequalities are also seen in neonatal healthcare and are more stark in the less well-resourced areas (8).

In 2015, the WHO published recommendations on interventions to reduce preterm mortality. Antenatal corticosteroids (ANS), CPAP and SRT have been identified as high impact interventions that improve the outcomes of preterm infants born in LMICs (9). However, the availability of these interventions has been low in most LMICs as several barriers limit their successful implementation.

The aim of this study was to describe the characteristics, respiratory interventions, and outcomes of all infants <1,801 g admitted to Groote Schuur Hospital (GSH) and Mowbray Maternity Hospital (MMH) during a 6-month period. The authors then extrapolated data from this study and estimated the mortality of these infants should interventions such as MV, SRT and CPAP not have been available.

## Materials and Methods

### Study Setting

GSH and MMH in Cape Town, South Africa are government funded institutions that serve a large indigent urban and peri-urban population within Cape Town. They provide tertiary and secondary level care, respectively, for a drainage area of 40,000 deliveries per annum. Although these public hospitals are lower resourced than their private for-profit counterparts in South Africa, they are better resourced than most other units in Africa (6).

GSH neonatal unit has 75 neonatal beds (20 ICU beds) and admits over 2,000 infants per year of which over 500 are very low birth weight (VLBW) (≤ 1,500 g). MMH, a dedicated maternity hospital with over 11,000 deliveries per year, has 73 neonatal beds (6 ICU beds) and admits over 2,500 infants per year of which 250 are VLBW. Hospital guidelines require all infants <1,801 g to be admitted routinely to these units.

The units offer intensive care interventions and support for a variety of common neonatal illnesses including RDS and sepsis. Preterm infants with respiratory distress are stabilized in the delivery room and transferred to the unit on CPAP. All infants admitted to these units are preferentially managed with a non-invasive ventilation strategy and inborn infants ≥ 27 weeks and ≥800 g have access to invasive ventilation options and SRT (8). On admission most preterm infants with respiratory distress are managed on flow-driver CPAP with 100 mg/kg of bovine surfactant administered *via* the LISA method should the oxygen requirements persistently exceed 40%. A repeat dose of surfactant may be administered after 6 h. Invasive Mechanical Ventilation (MV) includes both CMV and HFOV and may be used as the initial method of respiratory support for the very unwell infant, or for those who fail CPAP. Indications for MV include persistent FiO2 > 40% despite optimized CPAP and SRT (if indicated) or repeated apnoeas not responding to CPAP and caffeine therapy. CPAP failure is defined as the need for early MV, or death on CPAP. HHHF is available but is usually used as step down therapy after CPAP or ventilation. Due to resource constraints, infants born <800 g are not eligible for many of the interventions usually given for RDS in well-resourced areas. However, should they survive until they are more mature, they may become eligible to receive CPAP or MV later. Unlike many units in LMICs, continuous saturation monitoring and oxygen blenders are available for each infant as well as access to retinopathy of prematurity (ROP) screening, all of which help to reduce the incidence of ROP (10).

### Study Design

A 6-month prospective observational study was performed from 24 February to 23 August 2020. All infants <1,801 g admitted to GSH or MMH were included. Infants weighing <400 g and infants admitted to these units after 28 days of life were excluded. Infants were followed up until death or discharge home. Where available, gestational age was estimated from an early antenatal ultrasound (<20 weeks of gestation). Foot length or a New Ballard Score was performed for those remaining infants who did not have an early antenatal ultrasound (11). BPD was defined as an ongoing need for supplemental oxygen at 36 weeks corrected gestational age in infants born at <33 weeks.

### Data Collection

Data were collected onto REDCap (Research Electronic Data Capture) three times per week by a dedicated research officer. Data fields from the Vermont Oxford Network database were included, as well as additional demographic and outcome data (12). There was a particular focus on respiratory support requirements and surfactant administration.

### Statistical Analysis

Data were analyzed using STATA 15.1 (STATA Corporation, College Station, TX USA). Summary statistics included median (inter quartile range) for continuous variables and frequency (percent) for categorical variables. Statistical differences between groups were determined by chi-squared test of equal proportions and univariate logistic analysis. Kaplan-Meier survival analysis was used to determine differences in survival between infants <1,000 and ≥ 1,000 g. Crude incidence rates were determined for differences between groups; all statistical tests are two-sided (α = 0.05).

### Modeling Methodology

**Figure 1:**
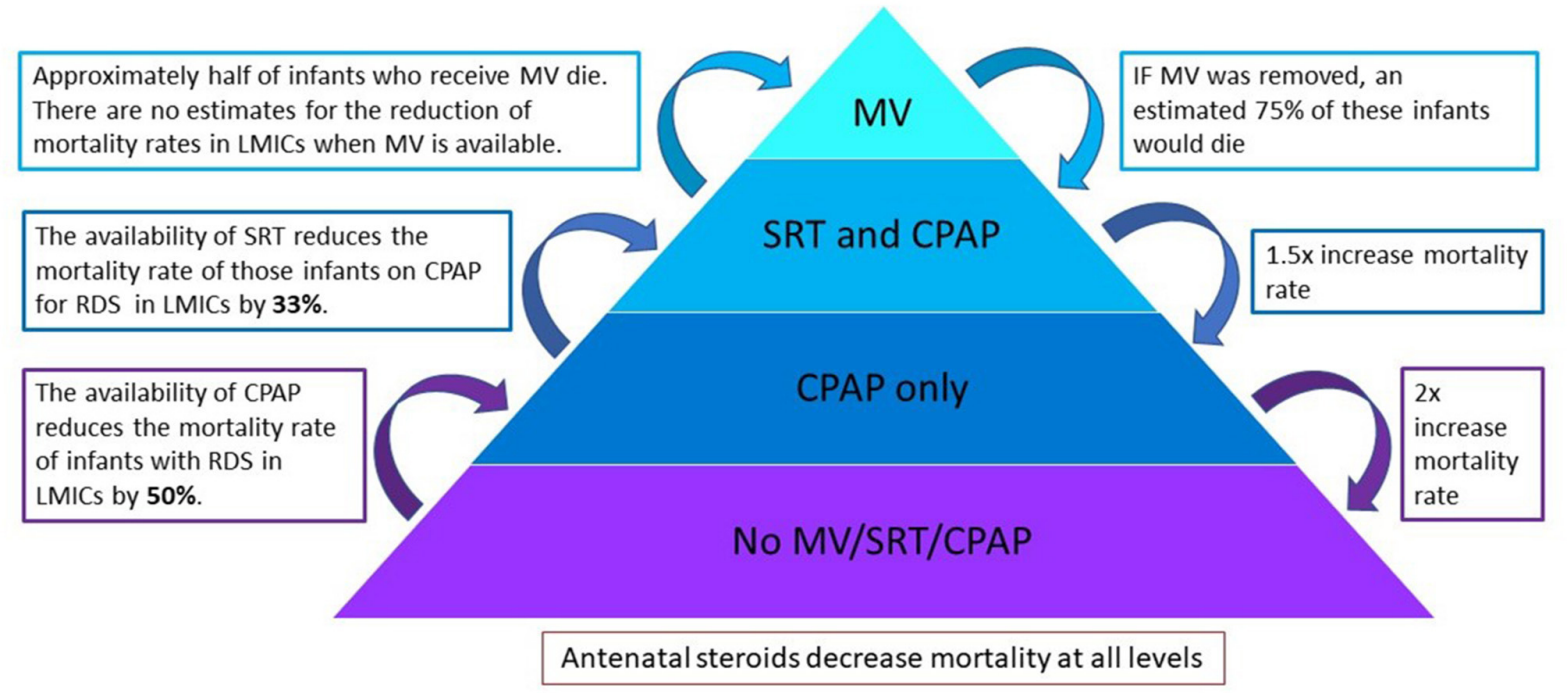
A pyramid composed of levels of interventions to illustrate the modeling methodology. Interventions are removed in a stepwise manner and the number of additional deaths is calculated at each subsequent step. MV, invasive mechanical ventilation; SRT, surfactant replacement therapy; CPAP, continuous positive airway pressure.

A model to estimate the number of additional deaths in the absence of available respiratory interventions (MV, SRT, and CPAP) was extrapolated from data of the observational study. The model was based on evidence from studies in LMICs on the efficacy of SRT and CPAP to reduce mortality. As seen in Figure 1, a pyramid composed of levels of interventions has been used to illustrate the modeling methodology. Interventions were removed in a stepwise manner and the number of additional deaths were calculated at each subsequent step. At the highest level, or top of the pyramid, all respiratory support interventions are available, and the overall mortality is that found in the observational study. Each subsequent removal of an intervention results in additional deaths and an increase in the overall mortality. At the lowest level, or base of the pyramid, no interventions are available, and this area represents a scenario where mortality is highest.

As there is limited available data on the reduction of mortality when MV is used in a LMIC setting, this was conservatively estimated based on the results from these units. After MV is removed, infants who had received both MV and SRT move to the level of SRT and CPAP, whilst those that did not receive SRT move to the level of CPAP only.

Calculations used based on published literature are estimated as follows:

Received MV—~50% of infants who receive MV in LMICs die (13, 14). It was therefore conservatively estimated that without MV 75% of infants from this group would demise.

Received SRT—SRT reduces the mortality rates of infants with RDS in LMICs by 33% (RR 0.67; 95% CI 0.57-0.79) (15). Removing SRT would therefore result in a 1.5 × increase in mortality rate in the SRT and CPAP group.

Received CPAP—CPAP reduces the mortality rate of infants with RDS in LMICs by 50% (16, 17). Removing CPAP would therefore result in a 2 × increase in mortality rate in the CPAP only group.

### Ethics Statement

This study was approved by the Human Research Ethics Committee, University of Cape Town and was performed in accordance with the Declaration of Helsinki. Individual informed consent for data collection was waived as: (i) this was an observational study with no identifying information collected; (ii) an incomplete database of this type would not be useful; and (iii) it was deemed insensitive to approach parents whose babies were extremely unwell or had died. Notices were displayed in visible areas within these units' informing mothers of potential data gathering for research purposes and parents could opt out of the data collection.

## Results

A total of 566 infants were included. There were 14 (2.5%) delivery room deaths all of whom either weighed <700 g or had life threatening congenital anomalies, resulting in a total of 552 infants admitted. Of the 552 infants, 366 (66.3%) were admitted at GSH and 186 (33.7%) were admitted at MMH. A total of 81 infants (14.7%) were outborn.

### Maternal Characteristics

Four hundred and ninety-three (89.3%) mothers attended at least one antenatal visit. Three hundred and eighteen (74.3%) mothers <34 weeks gestation received at least one dose of ANS, but one quarter of these received ANS more than 1 week before delivery. One hundred and thirty-seven mothers (43%) received only one dose of which 91 (28.6%) delivered prior to the optimal 24 h before delivery. Only 55 (17.3%) infants <34 weeks had optimal ANS (two doses completed between 24 h and 7 days before delivery).

Maternal infections included HIV (20.4%) and syphilis (3.6%). Birth by Cesarean Section occurred in 60.3% of cases with the main indications being concerns about fetal heart rate patterns on cardiotocography (36.2%) and maternal reasons (27.5%). Maternal hypertension was present in 43.6% of mothers.

### Neonatal Characteristics

Of the 552 infants, 258 (46.7%) were male and 126 (22.8%) were part of a multiple gestation. The median gestational age was 31 weeks (IQR 29-33 weeks). Gestational age was assessed using antenatal ultrasound in 32%, Ballard score in 31%, foot length in 22% and other/unknown in 15%.

**Figure 2:**
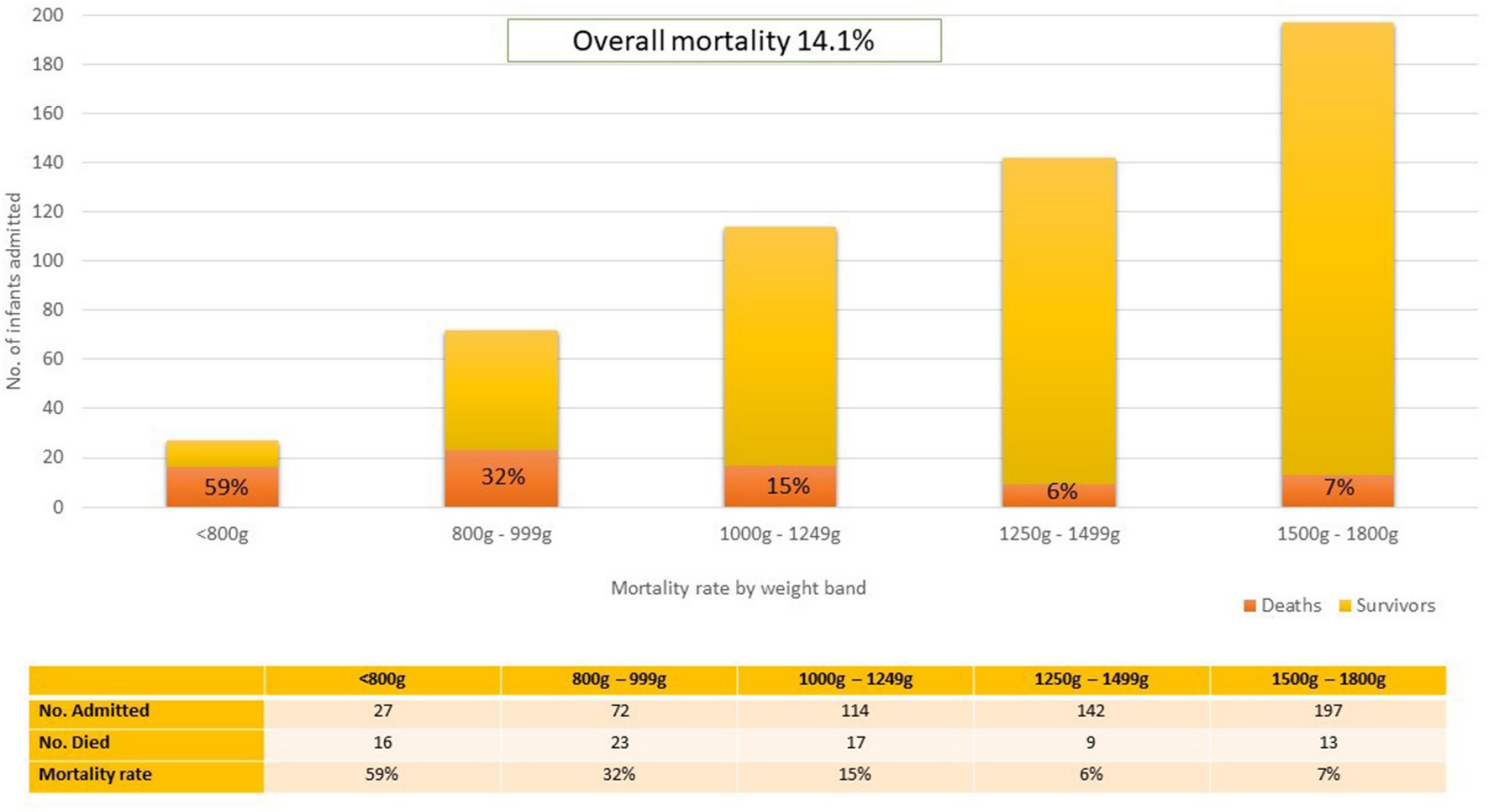
Mortality rate stratified by weight band. The total number of infants in each weight band has been depicted to illustrate the relative burden of admissions per weight band and deaths is depicted as a proportion of the total number of admissions.

The admission numbers and mortality rates per weight band are shown in Figure 2. The overall mortality rate for the cohort was 14.1%. Of the 78 total deaths, 11 (14.1%) had life threatening congenital anomalies (chromosomal or structural). Due to the severity of the anomalies, these infants were categorized as unavoidable deaths and were not offered respiratory support except for supplementary oxygen. Of those who demised, 34 infants (43.5%) died within the first 72 h and 6 (7.7%) died after transfer to other hospitals. In the first four weight categories each 250-gram gain in birth weight resulted in an approximate 50% reduction in mortality rate.

**Figure 3:**
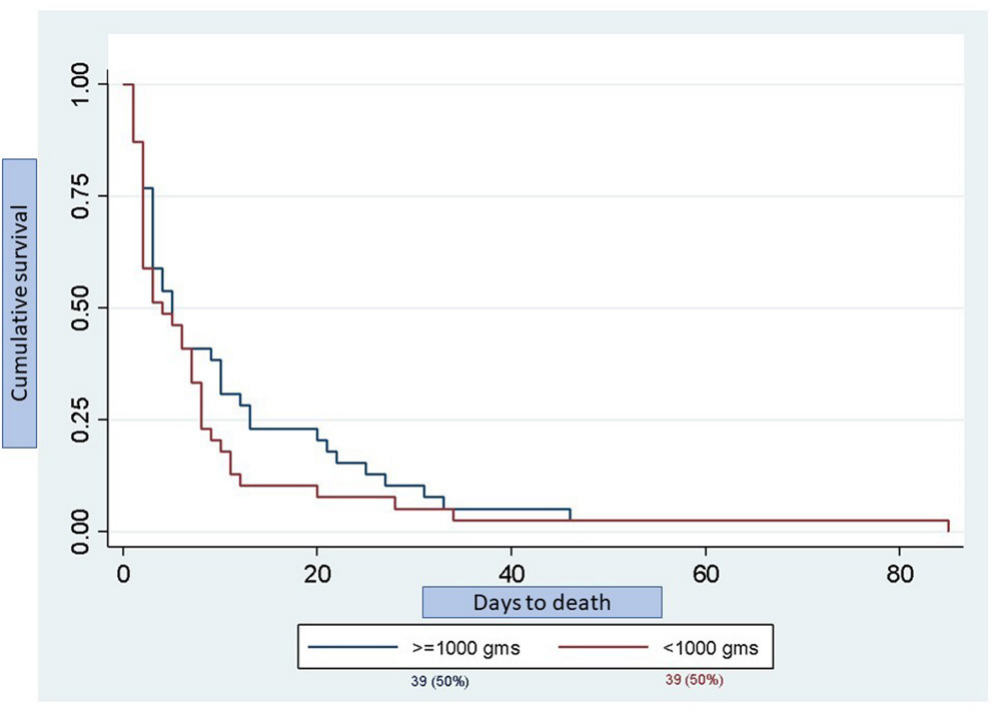
Kaplan-Meier graph depicting day of death stratified by birth weight. Total number of deaths was 78 infants of which 39 infants (50%) were <1,000 g and 39 infants (50%) were ≥1,000 g. There was no significant difference in the time to death between groups (*p* = 0.242).

Half (50%) of the deaths occurred in infants <1,000g and the time to death is shown in Figure 3. There was no significant difference in the time to death between infants <1,000 g and those ≥1,000 g (*p* = 0.242). There was also no significant difference in mortality between inborn (13.6%) and outborn (16.0%) infants (*p* = 0.35), or between singleton (15.5%) and multiple (16.1%) births (*p* = 0.17). The median weight for infants <34 weeks in both the steroid exposed and steroid unexposed groups was 1,390 g. A univariate logistic regression revealed that exposure to any ANS was associated with a 73% reduction in mortality (RR 0.27, 95% CI 0.15-0.45, *p* = 0.0001).

**Figure 4:**
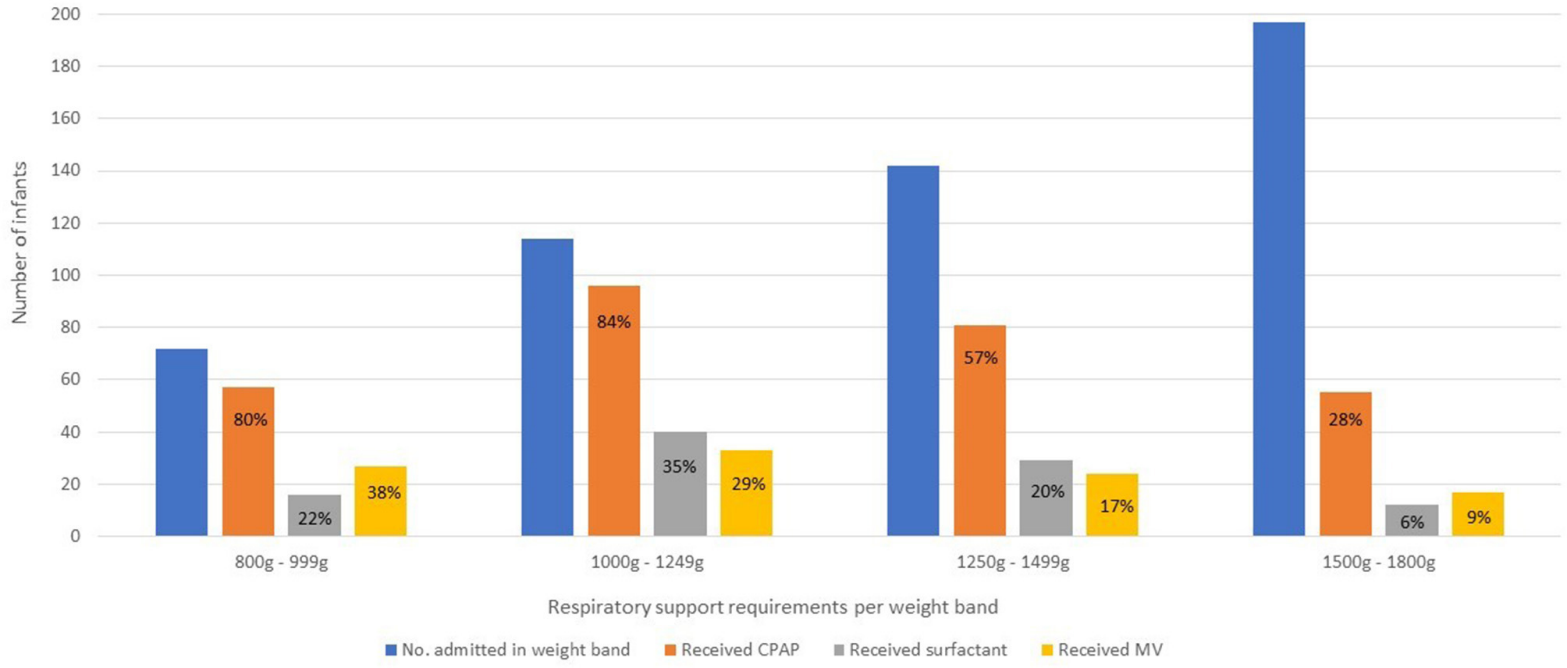
Respiratory interventions at any time during admission stratified by weight band. Due to resource constraints, infants born <800 g are not eligible for many of the interventions usually given for RDS in well-resourced areas. However, should they survive until they are more mature, they may become eligible to receive CPAP or MV later. CPAP, continuous positive airway pressure; MV, invasive mechanical ventilation.

The respiratory interventions given at any time during admission is shown by weight band in Figure 4.

### Continuous Positive Airway Pressure

Three hundred (54.3%) infants received CPAP, and this was the initial respiratory intervention for most cases of RDS. One hundred and fifty-three (82.3%) infants between 800 and 1,250g received CPAP. CPAP failure rates for RDS was 13%.

### Surfactant Replacement Therapy

Surfactant was administered to 100 (18.1%) infants and 27 (27%) of these infants received a second dose. Only 12 (6%) infants between 1, 500 and 1,800g received surfactant. Complications of administration occurred in 23 (23%) cases across all weight bands and included desaturation and/or bradycardia during surfactant administration (18), pulmonary hemorrhage (2), pneumothorax (1) and laryngospasm (1). LISA was the most common method for first dose administration (63%), followed by administration to infants already on a ventilator (21%). LISA was attempted without sedation and was successful in all but one infant who required intubation with an endotracheal tube.

### Invasive Mechanical Ventilation

One hundred and five (19%) infants received MV during their hospital stay but only 34 (9.1%) infants received ventilation as the initial method of respiratory support. CMV alone was used in 39 (37%) infants, HFOV alone in 13 (12%) and 53 (51%) infants received both CMV and HFOV. Infants were ventilated for RDS in the first few days of life, or subsequently for non-RDS reasons such as apnoea, sepsis, necrotising enterocolitis (NEC), or surgery. Only 57 (54.2%) of all ventilated infants survived until discharge from hospital, although infants ventilated for RDS only had better survival rates (68.4%).

### Other Respiratory Care

Oral caffeine was administered to 366 (98.9%) infants <33 weeks gestation. Thirty-two (6.7%) infants still required supplemental oxygen on day 28 of life, 4 (1.3%) infants received dexamethasone for BPD while only 6 (2%) infants born <33 weeks met criteria for BPD.

### Model Estimates

**Figure 5:**
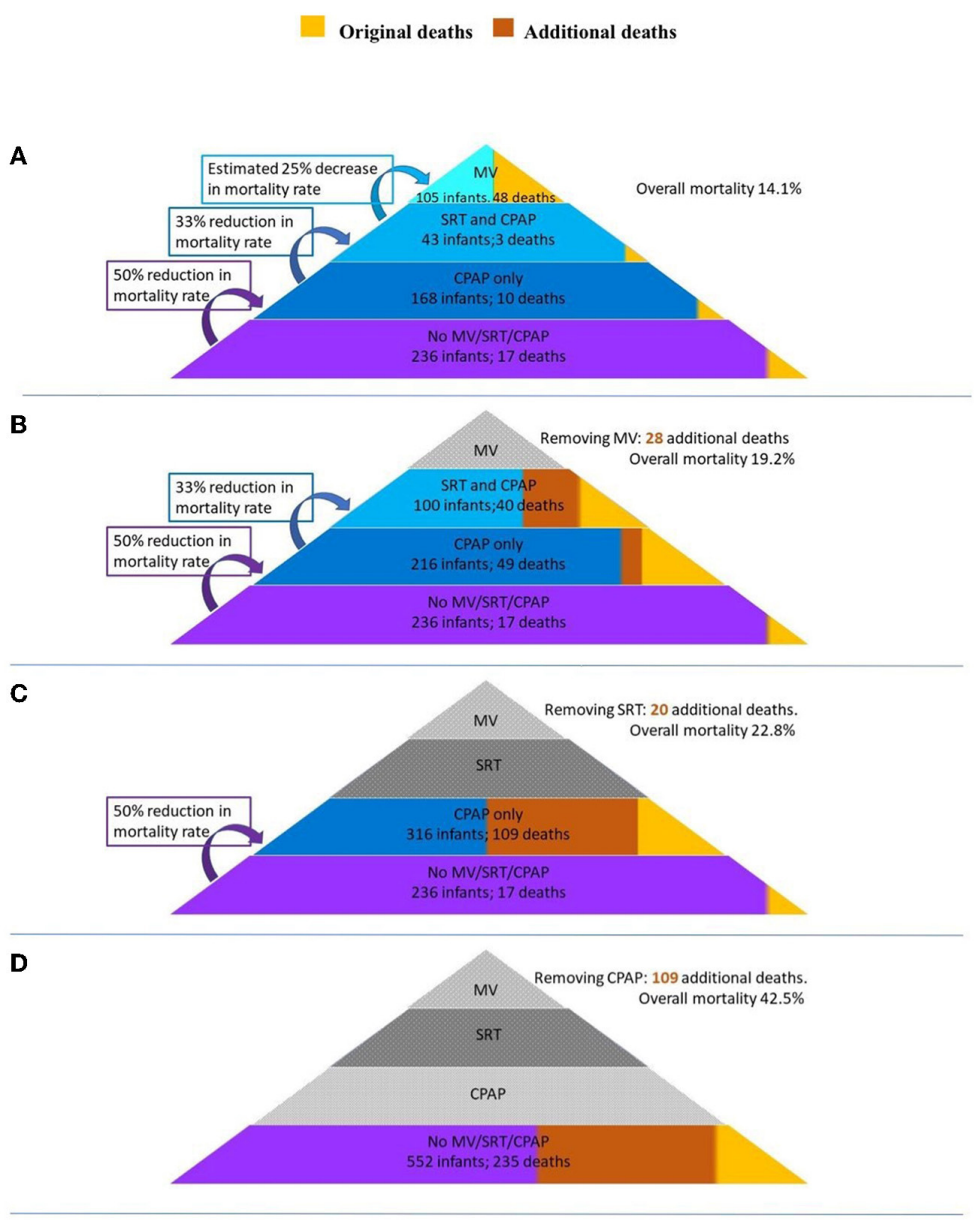
A model of the additional deaths and mortality rates of the study should respiratory interventions be sequentially removed. **(A)** All interventions available (baseline). **(B)** MV removed. **(C)** SRT removed. **(D)** CPAP removed. MV, invasive mechanical ventilation; SRT, surfactant replacement therapy; CPAP, continuous positive airway pressure.

Results from the model are illustrated in Figure 5. The removal of MV would result in 28 additional deaths and increase the overall mortality from 14.1 to 19.2%. The subsequent removal of SRT would result in a further 20 additional deaths and increase the overall mortality to 22.8%. Finally, the removal of CPAP would result in 109 additional deaths. The combined removal of MV, SRT, and CPAP would result in an additional 157 deaths increasing the overall mortality of the cohort to 42.5%. Of these 157 additional deaths, 50 would be in <1,000g weight band (increasing mortality from 39 to 90%), 84 would be in the 1,000-1,500 g weight band (increasing mortality from 10 to 43%) and 23 in the 1,500-1,800 g weight band (increasing mortality from 6.5 to 18.2%). A detailed description of the calculations can be found in Supplementary Figure 1.

## Discussion

This study is one of only a few funded prospective studies in a LMIC looking in detail at respiratory interventions and mortality in preterm infants. The mortality rate for each weight band is approximately double what may be found in large international databases such as the Vermont Oxford Network, and factors such as inadequate prenatal care, resource restrictions and higher patient to nursing ratios may contribute to these differences (12, 17). However, the mortality rate compares favorably to many other LMICs' results (2, 18–20). Although they serve a similar population to that found in many low-resourced areas, GSH and MMH neonatal units are much better resourced than many government institutions in LMICs (6).

To the best of the authors knowledge, this study is also one of the first to model changes in mortality rate if certain interventions were not available. It was estimated that mortality rates would triple from 14.1 to 42.5% without the combined availability of MV, SRT, and CPAP. Most of the additional deaths would occur in the 1,000-1,500 g weight band. Although these mortality rates are calculated specifically for GSH and MMH, these results might be extrapolated to other LMICs depending on the availability of resources in these countries. However, the availability of other life-saving interventions such as ANS, caffeine, and total parenteral nutrition as well as infection control and management are also important considerations. Furthermore, the incidence of maternal infections including HIV are important considerations although a recent study conducted in our setting showed no difference in mortality between HIV-exposed and HIV unexposed VLBW infants (21).

The use of ANS, supplemental oxygen with non-invasive and invasive ventilation options as well as SRT has dramatically improved survival rates of preterm infants, especially in High-income countries (HICs) (22). However, RDS remains one of the most important causes of neonatal mortality in LMICs. The availability of appropriate interventions and skills available to treat infants with RDS in LMICs is variable depending on where the infant is born (17). Many LMICs have limited or no access to many of these life-saving interventions leaving those infants that need it most even more vulnerable (23). Preterm infants born in rural areas have additional challenges; large distances with inadequate transport services hinder early and appropriate referral to larger institutions that may offer CPAP. Where SRT and MV are available, all levels of staff must be trained in the appropriate use and maintenance thereof (17). Infrastructure must be supportive to aid the implementation and utilisation of effective interventions to have a meaningful impact on neonatal mortality.

Achievement of the Sustainable Development Goals (2015-2030) is dependent upon meeting the Every Newborn 2020 targets and milestones (24). To meet the proposed target of fewer than 12 neonatal deaths or stillbirths per 1,000 births there needs to be a significant scale-up of effective interventions that address major causes of newborn deaths. Lassi et al. performed an overview of studies on available interventions aimed at preventing deaths in newborn infants and summarised the findings into three categories: effective interventions (high quality evidence), promising interventions (moderate quality evidence) and insufficient evidence. These interventions take place over a continuum of care from preconception to the postnatal period (23). See Supplementary Table 1 for an summary of all the effective and promising interventions as set out in Lassi et al.'s review.

### Continuous Positive Airway Pressure

CPAP has been defined as a promising intervention to improve preterm survival (23). Due to the high proportion of neonatal deaths from RDS, the WHO has made a strong recommendation for the use of CPAP as an intervention to improve preterm birth outcomes (9). In HICs, non-invasive ventilation strategies such as CPAP have been adopted as the standard of care in treating sick preterm infants with respiratory distress, with the option of SRT and MV if required (25, 26). An updated 2020 Cochrane review of CPAP for respiratory distress in preterm infants found that CPAP reduced the risk of mortality by 48% (RR 0.52, 95% CI 0.32-0.87) in HICs. In addition, there was low quality evidence that CPAP reduces the need for MV by 36%. The authors caution that these results may not be applicable in settings where advanced neonatal care is not readily available (27).

Several studies from LMICs have shown that the introduction of CPAP has resulted in improved neonatal outcomes. An observational study from Uganda assessing the impact of low-cost bubble CPAP (bCPAP) in VLBW infants found a 44% reduction in mortality (RR 0.56, 95% CI 0.36-0.86, *P* = 0.01) (28). A non-randomized study from Malawi to determine the efficacy of low-cost bCPAP to treat newborns with respiratory distress compared with nasal oxygen found a 27% absolute improvement in survival. The benefit of CPAP was more pronounced in VLBW infants and infants with RDS and/or sepsis (29). Similarly, a randomized controlled trial in Tanzania comparing bCPAP to oxygen therapy for preterm infants with signs of respiratory distress demonstrated a 30% improvement in survival to discharge in infants treated with bCPAP (30). Thukral et al. conducted a systematic review to evaluate the efficacy and feasibility of CPAP in LMICs and found its use resulted in a 66% reduction (OR 0.34, 95% CI 0.14-0.82) in the risk of in-hospital mortality (25). Finally, the Maternal and Neonatal Directed Assessment of Technology (MANDATE) model, used to evaluate how WHO-recommended interventions influence mortality of preterm infants in Sub-Saharan Africa, found that CPAP is a single intervention with the greatest impact on preterm mortality. The model assumes a 50% efficacy for the treatment of RDS (16).

All except the most premature infants <26 weeks born at GSH and MMH have access to CPAP. From our estimations the hypothetical removal of CPAP resulted in the largest number of excess deaths (109), more than the removal of MV and surfactant combined (48). It is evident from both the research studies and our estimations that CPAP can have a dramatic impact on preterm mortality in a low-resource setting.

Despite the evidence that CPAP is an effective treatment modality in preterm infants with respiratory distress in LMICs, the availability and utilization of this promising intervention remains low in Africa (6, 16). Although universal availability of CPAP to treat respiratory distress in preterm infants in LMICs may lead to a decrease in mortality, the low-resource setting in which the intervention is being implemented needs to be considered. CPAP machines must be low-cost, durable, and easy to maintain. In addition, staff need to be trained to correctly utilize the CPAP device and identify patients that may benefit from its use. The widespread adoption of CPAP in LMICs cannot be sustainable if the infrastructure to aid its use is not supportive (17).

### Surfactant Replacement Therapy

Despite advances in non-invasive respiratory support for preterm infants, SRT remains a key aspect of treatment for infants with established RDS (31). A Cochrane review of surfactant use in preterm infants in HICs with RDS found a 32% reduction in mortality and 53% reduction in air-leaks (32). Similarly, pooled analysis from a review of studies from LMICs showed a significant decrease in mortality (33% reduction, RR 0.67; 95% CI 0.57-0.79) and air leaks (RR 0.51; 95% CI 0.29-0.90) (15). The WHO has made a strong recommendation for the use of this promising intervention (9). Despite being included in the WHO Essential Drug List, the coverage of surfactant is estimated to be <40% in African countries (6).

We estimated that the lack of available surfactant in our setting would have resulted in 28 additional deaths. To realize the full impact of SRT, it cannot be used in isolation and the combined lack of CPAP and SRT would have resulted in the loss of nearly four times more (109) newborn lives (33).

In LMICs, lower limits for weight and gestational age for SRT should be carefully considered due to clinical and ethical concerns. Unacceptably high rates of BPD, intraventricular haemorrhage (IVH) and the need for MV may occur in extremely low birth weight (ELBW) infants (≤ 1,000 g) who require SRT and/or MV (15, 33, 34). The low incidence of BPD in infants born <33 weeks (2%) in our study may in part be explained by a local peri-viability management protocol which limits invasive ventilation in infants ≤ 27 weeks and ≤ 800 g.

The true benefit of SRT in LMICs is limited by the cost of the drug and the availability of skilled personnel and optimal respiratory support (15). However, the cost of surfactant is once-off compared to CPAP which requires maintenance and ongoing training of nursing staff. Further research into less- and non-invasive methods of SRT such as administration by laryngeal mask airway and aerosolization is needed.

### Invasive Mechanical Ventilation

There is limited available data on the efficacy of MV for preterm infants with respiratory distress in LMICs, but mortality rates for ventilated infants are estimated to be around 45-55% (13, 14). When available, its judicious use is often reserved for critically unwell larger infants as survival rates improve with increasing birth weight and gestational age (13). It has likely not been identified as an impactful intervention to reduce preterm mortality in LMICs due to significant barriers to its implementation including costs, maintenance, and sufficient healthcare providers skilled in intensive care. Of all the interventions, MV has the highest potential for harm, especially when performed poorly. Our estimates indicate that the lack of MV would have resulted in an additional 28 deaths in our cohort. Access to MV does potentially play a role in improving the outcomes of preterm infants in settings where advanced neonatal care is readily available.

### Antenatal Corticosteroids

The use of ANS is an important intervention for the prevention of RDS and it is the standard of care for the treatment of preterm birth in high resourced countries (35, 36). A 2017 Cochrane review found that treatment with ANS is associated with a reduction in the most serious adverse outcome associated with preterm birth including perinatal death, neonatal death, RDS, IVH, NEC and need for MV (37). It is important to note that most of the studies included in the review were conducted in HICs where there is universal coverage of intensive care, MV, and SRT for all infants. For this reason, the generalizability of this evidence in low resource countries has been called into question (35, 38). The benefit of ANS use in weak healthcare systems with inadequate support for preterm deliveries and care of the preterm infant has been a topic of debate (38). In addition, a large population-based Antenatal Corticosteroid trial (ACT) trial done in six LMICs found that ANS was associated with an increase in neonatal mortality and stillbirths (39).

The findings of the ACT trial and ongoing uncertainty about the use of ANS in LMICs resulted in further trials and reviews of studies done in LMICs. An updated Cochrane Review in 2020 found that treatment with ANS is associated with a reduction in perinatal deaths (15%), neonatal deaths (22%) and RDS (29%) (40). In contrast to the 2017 review that included studies from mostly HICs, this review included studies from a range of healthcare systems and resource settings including 10 from LMICs. Another subgroup meta-analysis of four studies in MICs found high-quality evidence of a substantial mortality effect (53%) of ANS and this effect is greater in MICs than in high-income settings (35). The recent WHO Action Trial, a multi-country randomized controlled trial found the use of ANS among women in LMICs who were at risk for early preterm birth resulted in significantly lower risks of neonatal death (36).

The WHO recommends the targeted use of ANS for women at risk of preterm birth with provision for adequate maternal and newborn care (41). Estimates on the use of ANS in LMICs vary greatly (10-41%) but despite the recommendation the coverage of ANS remains low in Sub-Saharan Africa and South-East Asia. Nearly three quarters of mothers in our study received at least one dose of ANS, but <20% received the recommended two doses more than 24 h but <7 days prior to delivery. Roberts *et al*. found that even one dose of ANS given <24 h before delivery reduces mortality of preterm infants by 47% (37).

A limitation of the study was that we did not attempt to model the impact of the absence of ANS in our setting because the ANS rate (variation in dose frequency and timing) was difficult to quantify (17.3-74.3%). Furthermore, the potential synergistic effects of ANS together with CPAP and SRT would make it difficult to predict the impact without ANS in isolation. The use of ANS in LMICs to reduce mortality in preterm infants is an important consideration. The model to estimate the number of additional deaths in our setting uses all-cause mortality as an outcome and was based on findings from published literature about LMICs. The findings of this study should only be considered as good estimates, and these estimates would need additional modifications in other settings.

## Conclusion

Preterm birth is the leading cause of neonatal mortality worldwide, with the highest burden in LMICs. This study estimated that a lack of CPAP availability would result in the largest number of excess deaths and that mortality rates of preterm infants in our setting would triple from 14.1 to 42.5% without the combined availability of MV, SRT, and CPAP. The use of ANS, CPAP and SRT as single interventions has been shown to improve the outcomes of preterm infants born in LMICs. Combined interventions packaged as bundles of care have the greatest impact on preterm mortality. Despite the evidence, coverage of these interventions in LMICs remains low and reductions in neonatal mortality inadequate. Several barriers to the introduction and dissemination of these evidence-based interventions exist within LMICs. Participation by all levels of policy makers, clinicians and community workers alike need to occur to see the successful implementation of interventions where they matter most.

Morbidity-free survival should be the goal for LMICs to prevent undue stress on families and health systems as long-term complications of prematurity amongst survivors exert a heavy burden on families, society, and health systems. Treatment should initially be targeted at those infants with the best possible short- and long-term outcomes. In those countries who are very resource limited, the primary focus for postnatal respiratory care should be on first implementing and delivering effective CPAP before additional interventions are introduced.

## Data Availability Statement

The raw data supporting the conclusions of this article will be made available by the authors, without undue reservation.

## Ethics Statement

The studies involving human participants were reviewed and approved by Human Research Ethics Committee, Faculty of Health Sciences, University of Cape Town. Written informed consent from the participants' legal guardian/next of kin was not required to participate in this study in accordance with the national legislation and the institutional requirements.

## Author Contributions

IL, CP, NR, HJZ, and LT contributed to the conception and design of the study. CP and LT developed the database. IL and LT performed the statistical analysis. IL and LT wrote the first draft of the manuscript. All authors contributed to manuscript revision, read, and approved the submitted version.

## Funding

Funding for this study was obtained from the Bill & Melinda Gates Foundation (BMGF)—Grant No: INV-000843. The BMGF did not play a role in the collection, analysis, or presentation of these data. Prof. Heather J Zar is supported by the SA-MRC Unit on Child & Adolescent Health, University of Cape Town.

## Conflict of Interest

The authors declare that the research was conducted in the absence of any commercial or financial relationships that could be construed as a potential conflict of interest.

## Publisher's Note

All claims expressed in this article are solely those of the authors and do not necessarily represent those of their affiliated organizations, or those of the publisher, the editors and the reviewers. Any product that may be evaluated in this article, or claim that may be made by its manufacturer, is not guaranteed or endorsed by the publisher.

## Supplementary Material

Supplementary Figure 1 Figure of calculations for modelling estimates. MV, invasive mechanical ventilation; surf, surfactant; CPAP, continuous positive airway pressure; LMIC, low-and-middle income countries; RDS, respiratory distress syndrome.

Supplementary Table 1 Table of interventions to improve neonatal health and later survival. ^*^Indicating what works—the review found high quality evidence with the effect likely to be similar to research findings; ^**^Indicating what might work but needs more evidence—the review found moderate quality evidence with the effect expected to be similar to research findings, but with a possibility that it will be substantially different in the future; Interventions in bold are discussed in detail in this manuscript.
