# Supplementary figures and images for: Respiratory Interventions for Preterm Infants in LMICs: A Prospective Study From Cape Town, South Africa
Source: Front Glob Womens Health. 2022 Apr 6;3:817817. doi: 10.3389/fgwh.2022.817817 (PMC9019119; doi:10.3389/fgwh.2022.817817)

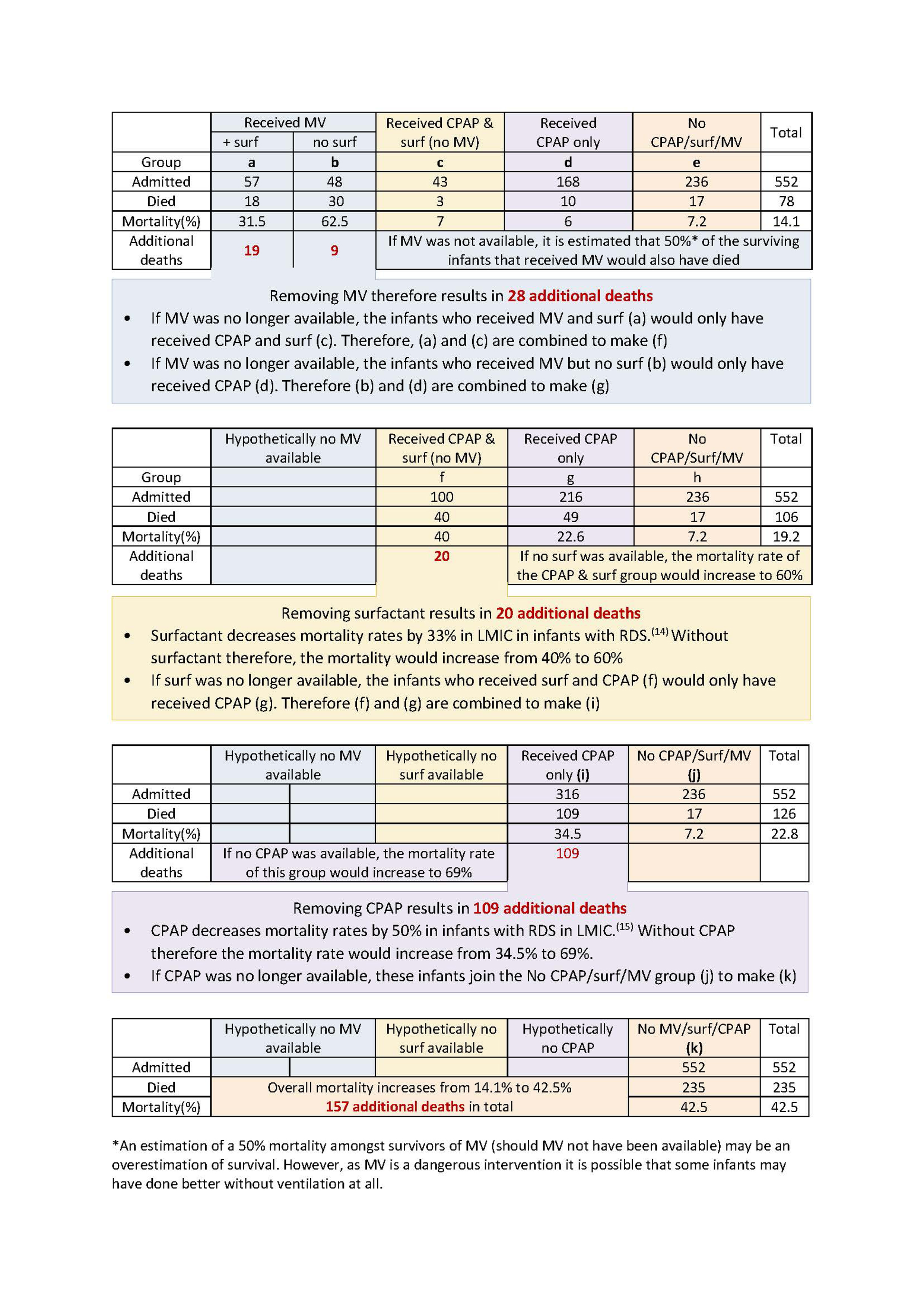

Supplement: Supplementary Figure 1 — Figure of calculations for modelling estimates. MV, invasive mechanical ventilation; surf, surfactant; CPAP, continuous positive airway pressure; LMIC, low-and-middle income countries; RDS, respiratory distress syndrome. [file Image_1.JPEG]
