# Supplementary material for: Respiratory Interventions for Preterm Infants in LMICs: A Prospective Study From Cape Town, South Africa
Source: Front Glob Womens Health. 2022 Apr 6;3:817817. doi: 10.3389/fgwh.2022.817817 (PMC9019119; doi:10.3389/fgwh.2022.817817)
Supplement: Supplementary Table 1 — Table of interventions to improve neonatal health and later survival. *Indicating what works—the review found high quality evidence with the effect likely to be similar to research findings; **Indicating what might work but needs more evidence—the review found moderate quality evidence with the effect expected to be similar to research findings, but with a possibility that it will be substantially different in the future; Interventions in bold are discussed in detail in this manuscript. [file Table_1.DOCX]

**Interventions to Improve Neonatal Health and Later Survival: An Overview of Systematic Reviews**

|  | *Effective Interventions | **Promising Interventions |
| --- | --- | --- |
| Antenatal intervention | **Corticosteroids for Preventing Neonatal Respiratory Distress Syndrome (RDS)** | Antenatal Care |
|  |  | Tetanus Immunization in Pregnancy |
|  |  | Prophylactic Antimalarials During Pregnancy |
|  |  | Provision and Promotion of ITNs During Pregnancy |
| Childbirth Interventions |  | Induction of Labour for Prolonged Pregnancy |
| Postnatal Interventions | Early Initiation of Breastfeeding | Case Management of Neonatal Sepsis, Meningitis and Pneumonia |
|  | Hygienic Cord Care | **Prophylactic and Therapeutic use of Surfactant** |
|  | Kangaroo Mother Care for Preterm Infants | **Continuous Positive Airway Pressure (CPAP)** |
| Child Health Interventions | Provision and Promotion of use of ITNs for Children | Case Management of Childhood Malaria |
|  | Vitamin A Supplementation From 6 Completed Months of age | Case Management of Childhood Pneumonia |
|  |  | Vitamin A as Part of Treatment for Measles-Associated Pneumonia for Children Above 6 Months |
|  |  | Home Visits Across the Continuum of Care women's Groups |

* Indicating what works – the review found high quality evidence with the effect likely to be similar to research findings.

** Indicating what might work but needs more evidence – the review found moderate quality evidence with the effect expected to be similar to research findings, but with a possibility that it will be substantially different in the future.

Interventions in **bold** are discussed in detail in this manuscript.

Reference: Lassi ZS, Middleton PF, Crowther C, Bhutta ZA. Interventions to improve neonatal health and later survival: an overview of systematic reviews. EBioMedicine. 2015;2(8):985-1000.
